# Epigenetic silencing of *Lgr5* induces senescence of intestinal epithelial organoids during the process of aging

**DOI:** 10.1038/s41514-018-0031-5

**Published:** 2018-12-01

**Authors:** Ryoei Uchida, Yoshimasa Saito, Kazuki Nogami, Yohei Kajiyama, Yukana Suzuki, Yasuhiro Kawase, Toshiaki Nakaoka, Toshihide Muramatsu, Masaki Kimura, Hidetsugu Saito

**Affiliations:** Division of Pharmacotherapeutics, Keio University Faculty of Pharmacy, 1-5-30 Shiba-kohen, Minato-ku, Tokyo, 105-8512 Japan

## Abstract

To understand the molecular features underlying stem cell aging, we established intestinal epithelial organoids derived from both young and aged mice and investigated alterations in their senescence and epigenetic status. Senescence-related changes including accumulation of senescence-associated β-galactosidase and up-regulation of *Cdkn1a* (*p21)* by DNA demethylation were observed in intestinal epithelial organoids derived from aged mice. We also demonstrated that the important stem cell marker *Lgr5* was epigenetically silenced by trimethylation of histone H3 lysine 27, inducing suppression of Wnt signaling and a decrease of cell proliferation in organoids from aged mice. We further treated intestinal epithelial organoids from aged mice with nicotinamide mononucleotide (NMN), a key NAD^+^ intermediate. As a result, the organoids showed a higher NAD^+^ level, increased cell proliferative ability, activation of *Lgr5* and suppression of senescence-associated genes, indicating that treatment with NMN could ameliorate senescence-related changes in intestinal epithelia. These findings suggest that organoids derived from aged animals could be a powerful research tool for investigating the molecular mechanisms underlying stem cell aging and for development of some form of anti-aging intervention, thus contributing to prolongation of healthy life expectancy.

## Epigenetic silencing of Lgr5 in aged organoids

To understand the molecular features underlying stem cell aging, we established intestinal epithelial organoids derived from both young and aged mice, and investigated alterations in their senescence and epigenetic status. Senescence-related changes including accumulation of senescence-associated β-galactosidase were observed in intestinal epithelial organoids derived from aged mice. We also demonstrated that the important stem cell marker *Lgr5* was epigenetically silenced by trimethylation of histone H3 lysine 27, inducing suppression of Wnt signaling and a decrease of cell proliferation in organoids from aged mice. Our results suggest that organoids derived from aged animals could be a powerful research tool for investigating the molecular mechanisms underlying stem cell aging and for development of some form of anti-aging intervention, thus contributing to prolongation of healthy life expectancy.

## Introduction

Cells are continuously exposed to aging-associated phenomena such as telomere shortening and oxidative stress.^1^ In various organs, homeostatic tissue maintenance and regenerative responses to injury depend on tissue-specific stem cells that have the capacity to both self-renew and differentiate into mature daughter cells. The life-long persistence of stem cells in the body makes them susceptible to accumulated cellular damage, which can finally lead to cell death, senescence or loss of regenerative function. Tissue-specific stem cells have been found to undergo changes with age, resulting in irregular responses to tissue injury, dysregulation of proliferation and decreased functional capacity, a phenomenon referred to as “stem cell aging”.^2^ Regulation of stem cell aging or age-associated stem cell dysfunction would be a key intervention for maximization of healthy life expectancy. However, in order to achieve this, it would be necessary to understand the molecular processes controlling stem cell survival, self-renewal, quiescence, proliferation, and commitment to specific differentiated cell lineages. Up to now, investigation of these aging-related processes has been difficult because of the lack of in vitro models that reflect the features of tissue-specific stem cells.

Lgr5, a member of the Wnt signaling pathway, has been identified as a new molecular marker of stem cells in endoderm-derived organs including the small intestine, colon, stomach, liver, and pancreas.^3–8^ The 3D culture system known as organoid culture allows long-term expansion of Lgr5-positive stem cells into cyst-like structures (organoids) with properties resembling those of the original tissues. This type of 3D culture uses serum-free medium that includes only defined factors such as R-spondin 1 (Rspo1), epidermal growth factor (EGF), and noggin. Rspo1 has been identified as a ligand for Lgr5 and an essential factor for activation of the Wnt signaling pathway.^9,10^ Using this 3D culture system, cancer and non-cancer organoids derived from human colon, prostate, pancreas, and liver have been established.^6,11–14^ We have also reported that inhibition of DNA methylation suppresses the growth of intestinal tumor organoids, and that when organoids derived from human intrahepatic cholangiocarcinomas are induced to differentiate to hepatocytes, their malignant potential is reduced.^15,16^

Thus, organoids derived from various tissues can be powerful research tools that reproduce their properties, including disease symptoms and response to therapeutics. Among human tissues, we have focused on intestinal epithelia to study stem cell aging. Since intestinal epithelial cells have a very short turnover time, stem cells in intestinal epithelia are a good model for investigations of stem cell function. In the present study using intestinal epithelial organoids derived from young and aged mice, we investigated the molecular mechanism underlying stem cell aging with a view to devising some form of anti-aging intervention.

## Results

### Senescence-related changes in intestinal epithelial organoids derived from aged mice

**Fig. 1:**
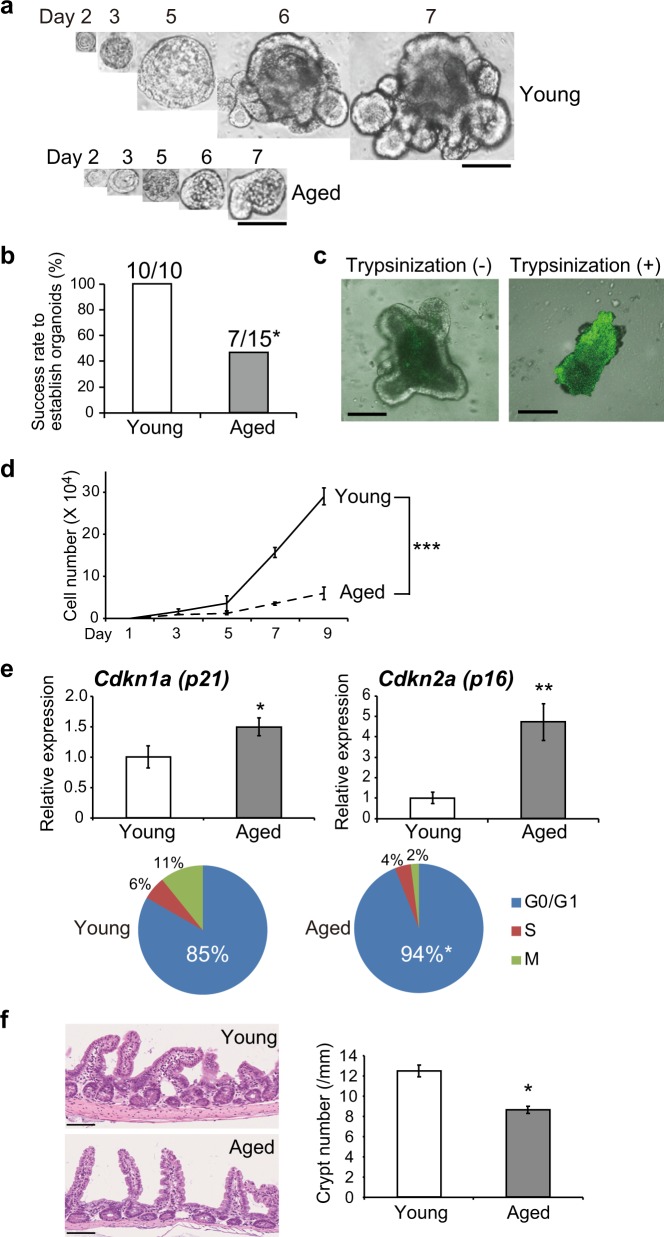
Senescence-related changes in intestinal epithelial organoids derived from aged mice. **a** Representative images of a single stem cell expanding into organoids derived from intestinal epithelia of young (4 weeks) and aged (54 weeks) mice. Scale bars: 250 μm. **b** Success rate of organoid establishment from young mice (4–27 weeks, *n* = 10) and aged mice (54–130 weeks, *n* = 15). We defined the establishment of organoids as successful if it was possible to culture them over 5 passages. *p < 0.05. **c** TUNEL assay for detection of apoptosis. Apoptotic cell death was observed in intestinal epithelial organoids after trypsin treatment without the Rho-kinase inhibitor Y-27632. Scale bars: 100 μm. **d** Cell numbers in intestinal epithelial organoids derived from young (4 weeks) and aged (54 weeks) mice during the culture course. Results of 5 experiments were plotted as mean ± SD. ***p < 0.001. **e** Relative expression levels of *Cdkn1a (p21)* and *Cdkn2a (p16)* and cell cycle analysis of intestinal epithelial organoids established from young (4 weeks) and aged (54 weeks) mice. Results of 5 experiments were plotted as mean ± SD. *p < 0.05, **p < 0.01. **f** The numbers of intestinal crypts in young (10 weeks) and aged (120 weeks) mice. HE staining of intestinal crypts in young and aged mice are shown. Scale bars: 100 μm. The number of intestinal crypts (/mm) was significantly decreased in aged mice in comparison to young mice. Results of 5 experiments were plotted as mean ± SD. *p < 0.05

We established organoids using intestinal epithelial tissues obtained from mice at various ages. Figure 1a shows representative images of a single stem cell expanding into organoids derived from intestinal epithelia of young (4 weeks) and aged (54 weeks) mice. We observed sequential formation of tissue-like structures from a single stem cell after trypsinization of the organoids. The organoids derived from young mice grew larger, forming typical structures resembling intestinal crypts with budding. On the other hand, some of the organoids derived from aged mice failed in the formation of crypt-like structures. The organoids derived from the aged mouse were smaller and could not be maintained for a long period (Fig. 1a). We further established organoids using intestinal epithelial tissues from a total of 25 mice at various ages. We considered establishment of organoids to be successful if it was possible to culture them over 5 passages, and compared the success rates for organoid establishment between mice aged more than 50 weeks and younger mice. As shown in Fig. 1b, the success rate of organoid establishment from aged mice (54–130 weeks, *n* = 15) was significantly lower than that from younger mice (4–27 weeks, *n* = 10). The proliferative ability of organoids established from young and aged mice was then examined by counting the number of cells obtained after organoid trypsinization. It has been reported that normal intestinal epithelial cells have a tendency to become apoptotic when they are isolated by trypsinization because of excessive myosin activation. To inhibit myosin activation, trypsinized cells were cultured with the Rho-kinase inhibitor Y-27632. As shown in Fig. 1c, apoptotic cell death was observed in intestinal epithelial organoids after trypsin treatment without Y-27632. The number of cells in intestinal epithelial organoids derived from young mice was significantly higher than that in organoids from aged mice (Fig. 1d).

Transcription of the senescence markers *Cdkn1a (p21) and Cdkn2a (p16)*, which are expressed in a coordinated manner with aging, was significantly increased in intestinal epithelial organoids established from aged mice (Fig. 1e). Flow cytometric analysis of the cell cycle showed that the population in G0/G1 phase was significantly elevated in intestinal epithelial organoids from aged mice (Fig. 1e). These findings indicate that intestinal epithelial organoids derived from aged mice showed changes suggestive of senescence.

A recent study has demonstrated that aging alters the architecture of the intestinal crypt and villus, and also cell proliferation, in vivo.^17^ It was found that the number of intestinal crypts was significantly decreased in aged mice in comparison to young mice. When we compared the numbers of intestinal crypts in young (10 weeks) and aged (120 weeks) mice (Fig. 1f), the number of intestinal crypts was significantly decreased in aged mice, being consistent with the results of our in vitro study using intestinal epithelial organoids.

**Fig. 2:**
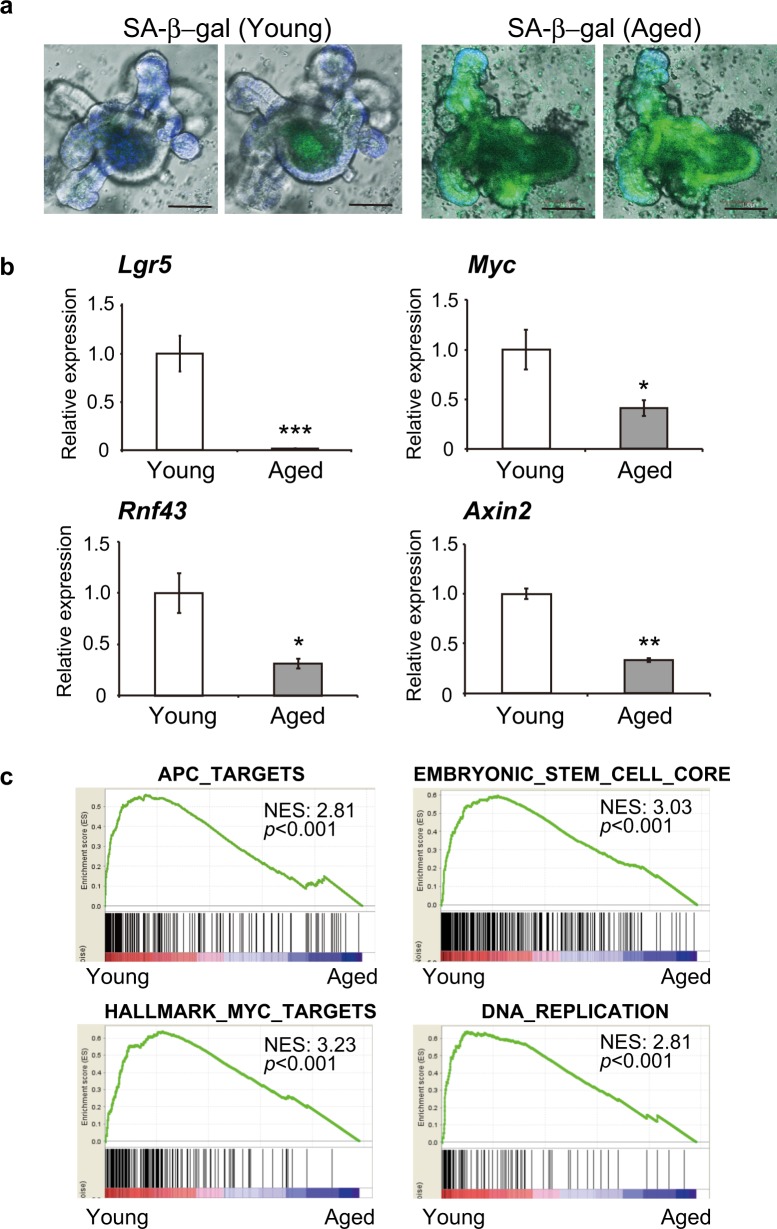
Suppression of *Lgr5* and the Wnt signaling pathway in intestinal epithelial organoids from aged mice. **a** Detection of SA-β-gal in intestinal epithelial organoids derived from young (6 weeks) and aged (78 weeks) mice. Accumulation of SA-β-gal was observed in aged intestinal epithelial organoids, whereas SA-β-gal was not detected in young organoids. Scale bars: 100 μm. **b** Relative expression levels of *Lgr5* and Wnt signaling pathway genes in intestinal epithelial organoids derived from young (4 weeks) and aged (54 weeks) mice. Results of 5 experiments were plotted as mean ± SD. *p < 0.05, **p < 0.01, ***p < 0.001. **c** GSEA with the gene sets APC_TARGETS, EMBRYONIC_STEM_CELL_CORE, HALLMARK_MYC_TARGETS and DNA_REPLICATION in intestinal epithelial organoids derived from young (4 weeks) and aged (54 weeks) mice

To obtain direct evidence of cellular senescence in aged intestinal organoids, we examined the presence of senescence-associated β-galactosidase (SA-β-gal) in intestinal epithelial organoids derived from young (6 weeks) and aged (78 weeks) mice. As shown in Fig. 2a, accumulation of the SA-β-gal was observed in aged intestinal epithelial organoids, whereas this was not evident in young intestinal epithelial organoids. These results, in addition to the expression of senescence markers (*p21* and *p16*) and findings from the cell cycle assay, indicate that intestinal epithelial organoids derived from aged mice can be used as an in vitro model of aging.

### Suppression of Lgr5 and the Wnt signaling pathway in intestinal epithelial organoids from aged mice

We next performed microarray analysis to identify genes that were differentially expressed between intestinal epithelial organoids derived from young and aged mice. Interestingly, the gene showing the greatest decrease of expression in intestinal epithelial organoids from aged mice (54 weeks) in comparison with those from young mice (4 weeks) was *Lgr5* (Table 1). Other stem cell markers, including *Olfm4*, were also down-regulated in intestinal epithelial organoids from aged mice.^18^ Using real-time PCR, we confirmed that the transcription of *Lgr5* was significantly decreased in intestinal epithelial organoids derived from aged mice (Fig. 2b). Lgr5 is the receptor responsible for triggering the Wnt signal cascade that induces transition of β-catenin into the nucleus. Nuclear β-catenin activates the expression of *Lgr5, Myc, Rnf43*, and *Axin2*, which then maintain cell proliferation. We examined the transcription levels of *Myc, Rnf43*, and *Axin2*, which are downstream in the Wnt signaling pathway, and confirmed that they were significantly decreased (Fig. 2b). We then performed gene set enrichment analysis (GSEA) using microarray data for intestinal epithelial organoids derived from young and aged mice using the gene sets APC_TARGETS, EMBRYONIC_STEM_CELL_CORE, HALLMARK_MYC_TARGETS and DNA_REPLICATION. As shown in Fig. 2c, GSEA demonstrated that the gene sets associated with the Wnt signaling pathway, stem cell signature and DNA replication were significantly suppressed in intestinal epithelial organoids established from aged mice. These results suggested that suppression of *Lgr5* induces inactivation of the Wnt signaling pathway and a decrease of the stem cell signature, resulting in cellular senescence and a decrease of cell proliferation in intestinal epithelial organoids derived from aged mice.

**Table 1:** Genes differentially expressed between intestinal epithelial organoids derived from mice aged 4 and 54 weeks

	Gene symbol	4 W	54 W	Ratio (54 W/4 W)
1	*Lgr5*	792.9	23.7	0.030
2	*Gpc3*	235.4	12.2	0.052
3	*Olfm4*	7856.0	416.4	0.053
4	*Slc28a2*	428.7	26.9	0.063
5	*Stc2*	112.3	8.4	0.075
6	*Anxa1*	126.2	9.6	0.076
7	*Rassf5*	115.4	9.6	0.083
8	*Clu*	224.0	18.9	0.084
9	*Nr2e3*	154.3	13.3	0.086
10	*Pla2g1b*	74.1	6.7	0.090

### Epigenetic silencing of Lgr5 in intestinal epithelial organoids from aged mice

**Fig. 3:**
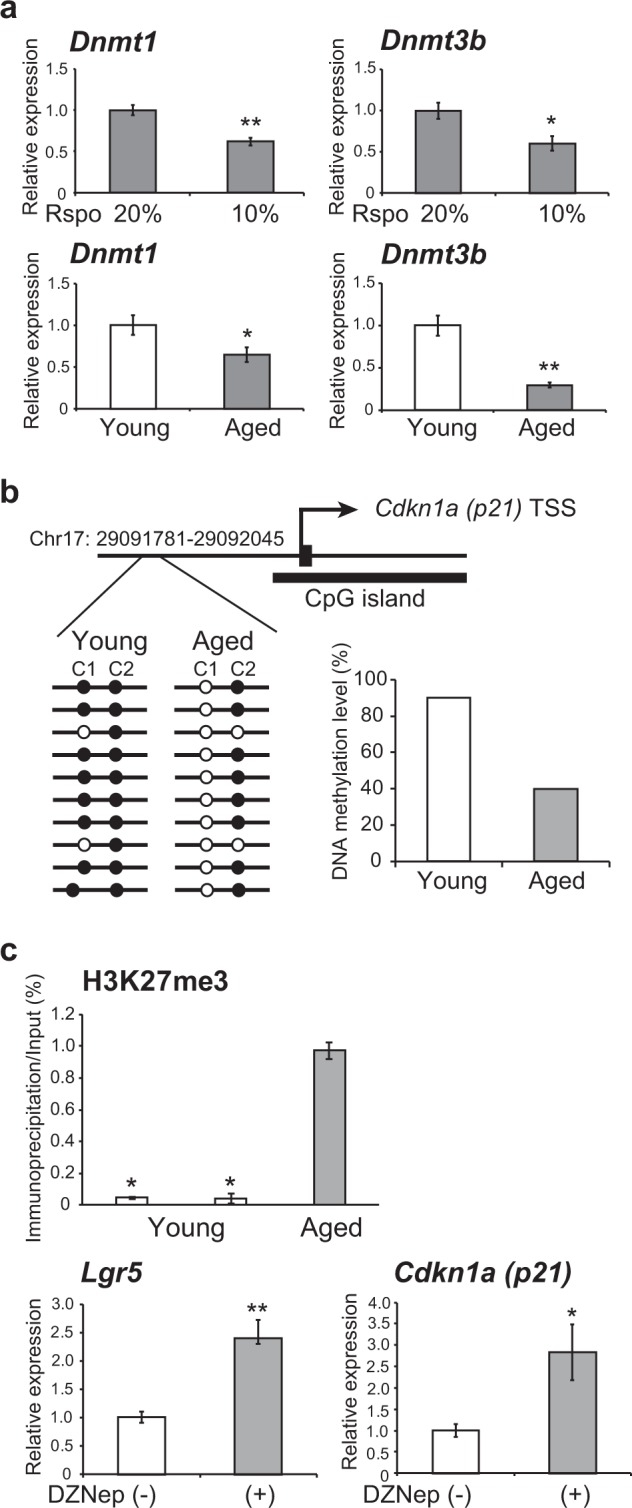
Epigenetic silencing of *Lgr5* in intestinal epithelial organoids from aged mice. **a** Relative levels of *Dnmt1* and *Dnmt3b* expression in intestinal epithelial organoids derived from young (4 weeks) mice after reducing the concentration of Rspo1 from 20 to 10% in the culture medium, and in intestinal epithelial organoids derived from young (4 weeks) and aged (54 weeks) mice. Results of 5 experiments were plotted as mean ± SD. *p < 0.05, **p < 0.01. **b** DNA methylation levels in the promoter region of the *Cdkn1a* (*p21*) gene in intestinal epithelial organoids derived from young (4 weeks) and aged (54 weeks) mice. The map shows the transcription start site (TSS) of the *p21* gene and the region in which DNA methylation levels were analyzed by bisulfite sequencing. Black circle, methylated CpG; White circle, unmethylated CpG. **c** ChIP assay for trimethylation on histone H3K27 in the *Lgr5* promoter region in intestinal epithelial organoids derived from young (27 weeks) and aged (60 weeks) mice. Relative expression levels of *Lgr5* and *p21* in intestinal epithelial organoids derived from aged (60 weeks) mice with or without exposure to DZNep for 7 days. Results of 5 experiments were plotted as mean ± SD. *p < 0.05, **p < 0.01

It has been reported that knockdown of the DNA methyltransferase *Dnmt1* induces up-regulation of *p21* by DNA demethylation in the promoter region in intestinal organoids.^19^ This prompted us to investigate epigenetic alterations in intestinal epithelial organoids from aged mice. We examined the effect of Wnt signaling suppression in intestinal epithelial organoids established from young mice by reducing the concentration of Rspo1, a ligand of Lgr5, in the culture medium. Reduction of the Rspo1 concentration by half significantly decreased the expression of the DNA methyltransferases *Dnmt1* and *Dnmt3b* (Fig. 3a). The levels of *Dnmt1* and *Dnmt3b* expression were indeed reduced in intestinal epithelial organoids established from aged mice in comparison to those from young mice (Fig. 3a). Using bisulfite sequencing, we then examined the DNA methylation pattern in the promoter region of the *p21* gene. As shown in Fig. 3b, some of the cytosine residues in the CpG islands of the *p21* promoter were demethylated in intestinal epithelial organoids from aged mice in comparison to those from young mice.

To investigate epigenetic regulation of *Lgr5*, we examined DNA methylation levels around the promoter region of the *Lgr5* gene, but no significant changes were evident in intestinal epithelial organoids derived from either young or aged mice (data not shown). Using the chromatin immunoprecipitation (ChIP) assay, we then examined histone modification of the *Lgr5* promoter region in intestinal epithelial organoids. Chromatin samples were prepared from intestinal epithelial organoids established from young and aged mice, and the levels of trimethylation on histone H3 lysine 27 (H3K27) were compared. The levels of trimethylation on histone H3K27 were significantly higher in intestinal epithelial organoids from aged mice than in those from young mice (Fig. 3c). We also examined the effect of the histone methyltransferase EZH2 inhibitor, 3-deazaneplanocin A (DZNep), on regulation of *Lgr5* expression. The levels of *Lgr5 and p21* expression in intestinal epithelial organoids from aged mice were significantly elevated after the exposure to DZNep for 7 days (Fig. 3c). These results suggest that epigenetic alterations, including silencing of *Lgr5* by trimethylation on histone H3K27, are an important event during stem cell aging.

### Effect of nicotinamide mononucleotide on senescence of intestinal epithelial organoids from aged mice

**Fig. 4:**
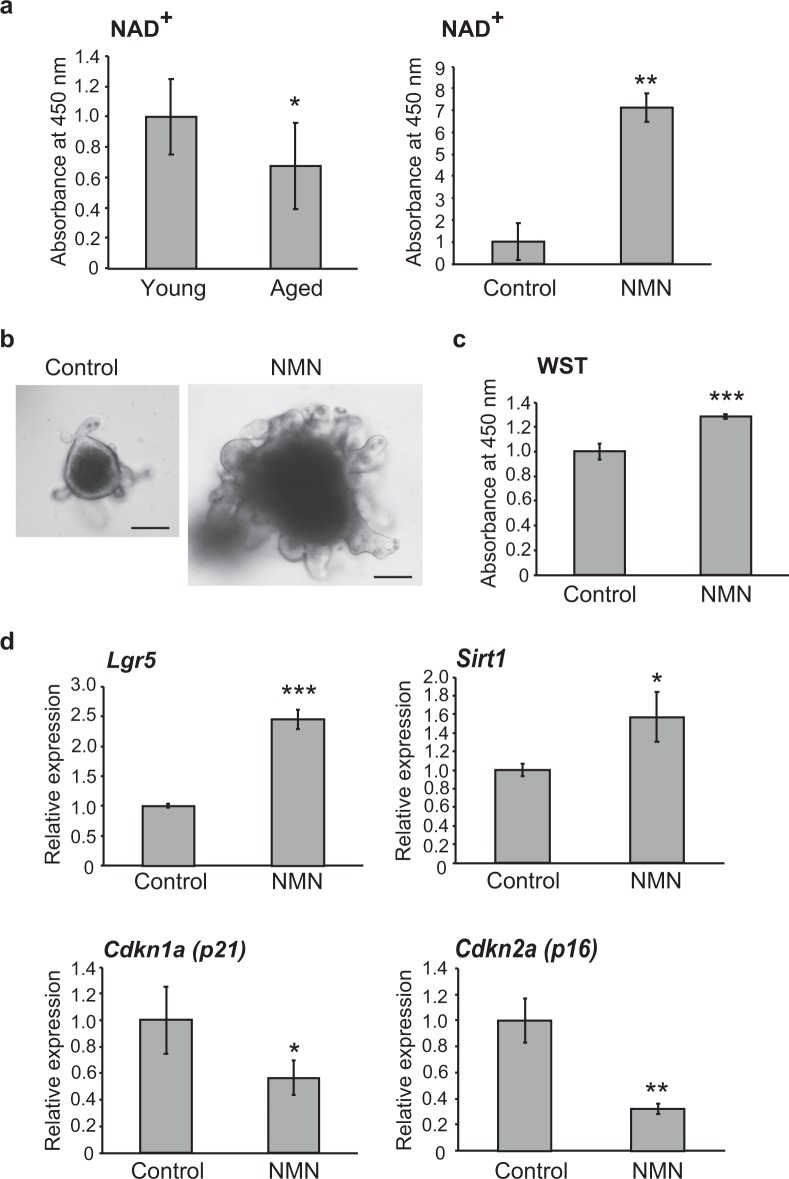
Effect of NMN on senescence of intestinal epithelial organoids from aged mice. **a** NAD^+^ levels of organoids derived from intestinal epithelia of young (7 weeks) and aged (55 weeks) mice (left). NAD^+^ levels of intestinal epithelial organoids derived from aged (76 weeks) mice with or without NMN treatment (right). Results of 8 experiments were plotted as mean ± SD. *p < 0.05, **p < 0.01. **b** Representative images of intestinal epithelial organoids derived from aged (76 weeks) mice with or without NMN treatment for 7 days. Scale bars: 100 μm. **c** WST cell proliferation assay of intestinal epithelial organoids derived from aged (76 weeks) mice with or without NMN treatment for 10 days. Results of 8 experiments were plotted as mean ± SD. ***p < 0.001. **d** Relative expression levels of *Lgr5, Sirt1, p21*, and *p16* in intestinal epithelial organoids derived from aged (76 weeks) mice with or without NMN treatment. Results of 5 experiments were plotted as mean ± SD. *p < 0.05, **p < 0.01, ***p < 0.001

Recent studies have reported that aging induces reduction of NAD^+^ in the body and that its supplementation induces longevity and stem cell activation.^20–23^ We measured NAD^+^ level in intestinal epithelial organoids derived from young and aged mice, and treated aged intestinal epithelial organoids with nicotinamide mononucleotide (NMN), a key NAD^+^ intermediate. As shown in Fig. 4a, the NAD^+^ level was decreased in organoids derived from aged mice, but significantly increased after treatment with NMN. Furthermore, the numbers of crypt-like structures showing budding and cell proliferation activity were significantly increased in aged intestinal epithelial organoids treated with NMN (Figs. 4b, c). In addition, gene expression profiling showed that *Lgr5* and *Sirt1* were up-regulated, and that senescence-associated genes such as *p21* and *p16* were suppressed after NMN treatment (Fig. 4d). These findings suggest that treatment with NMN increases the NAD^+^ level and may ameliorate senescence-related changes in intestinal epithelia.

## Discussion

Here we have demonstrated that the important stem cell marker *Lgr5* was epigenetically silenced by trimethylation of histone H3K27, inducing suppression of Wnt signaling and a decrease of cell proliferation in intestinal epithelial organoids derived from aged mice. In these organoids, we also observed accumulation of SA-β-gal, a decrease in the expression of DNA methyltransferases and an increase in the expression of *p21* accompanied by DNA demethylation in the promoter region. These dynamic epigenetic modifications led to a decrease of cell proliferation and dysfunction of stem cells, possibly resulting in aplasia and dysfunction of intestinal epithelia during aging.

**Fig. 5:**
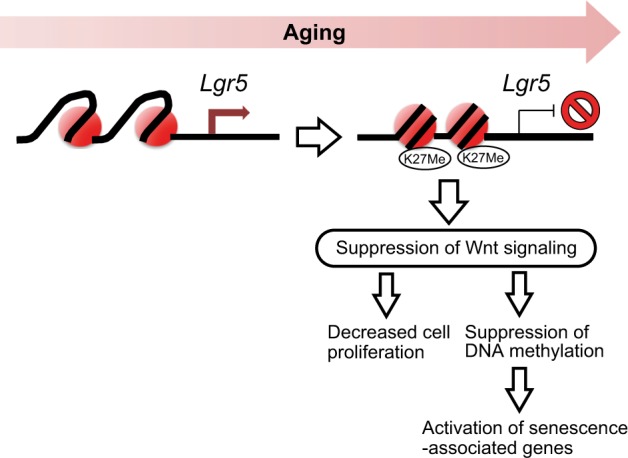
Possible mechanism for epigenetic silencing of *Lgr5* and induction of senescence in intestinal epithelial organoids derived from aged mice. The stem cell marker *Lgr5* is substantially expressed in young intestinal epithelial organoids, but is epigenetically silenced by histone H3K27 trimethylation in organoids from aged intestinal epithelium. Since Lgr5 is an activator of the Wnt signaling pathway, epigenetic silencing of *Lgr5* results in suppression of Wnt signaling, which may lead to decreased cell proliferation and activation of senescence-associated genes such as *p21* due to suppression of DNA methylation

Figure 5 shows a possible mechanism for epigenetic silencing of *Lgr5* and induction of senescence in aged intestinal organoids. The stem cell marker *Lgr5* was substantially expressed in young intestinal epithelial organoids, whereas it was faintly expressed in aged intestinal organoids. Examination of DNA methylation levels around the *Lgr5* promoter region revealed no significant difference in DNA methylation between young and aged intestinal organoids. It has been reported that a closed chromatin structure associated with trimethylation of histone H3K27 leads to silencing of gene expression independently of DNA methylation.^24,25^ The results of our ChIP assay indicated that increased levels of histone H3K27 trimethylation led to silencing of *Lgr5* expression in aged intestinal epithelial organoids. Since Lgr5 is an activator of the Wnt signaling pathway, epigenetic silencing of *Lgr5* results in suppression of Wnt signaling, which may lead to decreased cell proliferation and activation of senescence-associated genes such as *p21* due to suppression of DNA methylation. A recent in vivo study has shown that intestinal stem cell function is altered because of reduced canonical Wnt signaling upon aging, and that canonical Wnts are reduced in intestinal stem cells of aged mice.^17^ This study is consistent with the results we obtained for the Wnt signaling pathway in young and aged intestinal organoids.

Vincent et al. have developed an ex vivo method for studying the expression and epigenetic profiles of stem cells during their differentiation to epithelial cells by isolating cryosections of the intestinal crypt-villus axis.^26^ They found that *Lgr5* is highly expressed in crypts, but is silenced by trimethylation of histone H3K27 in the villi. In addition, a recent study has shown that knockdown of *Dnmt1* in intestinal organoids induced demethylation of the *p21* gene, leading to an increase of p21 expression, induction of cellular aging, and finally aplasia of crypt-like structures.^19^ These findings lend strong support to our present results and indicate that stem cell aging is epigenetically induced through inactivation of *Lgr5* and the Wnt signaling pathway.

Recently, calorie restriction experiments have highlighted *Sirt1* as a possible longevity gene.^27,28^ Sirt1 has histone acetyl transferase activity and its expression is regulated by the concentration of NAD^+^.^29^ Aging leads to a reduction of NAD^+^ in the body, and it has been reported that supplementation of NAD^+^ induces longevity and stem cell activation.^20,21^ Here, intestinal epithelial organoids derived from aged mice grew larger, forming crypt-like structures after treatment with NMN, a key NAD^+^ intermediate. The aged intestinal epithelial organoids treated with NMN showed an increase of proliferative activity, activation of *Lgr5* and *Sirt1*, and suppression of *p21* and *p16*, suggesting that treatment with NMN was able to ameliorate senescence-related changes in intestinal epithelia and could have potential application as an anti-aging intervention. We speculate that the NAD^+^-dependent deacetylase Sirt1 which may modulate directly or indirectly on the epigenetic alterations such as DNA methylation and histone modification around the promoter region of the *Lgr5* gene, although further studies will be necessary to reveal the molecular mechanism underlying epigenetic regulation of *Lgr5* in association with NAD^+^ in intestinal epithelia during the process of aging.

Thus, using organoids derived from intestinal epithelia of aged mice, we have demonstrated that epigenetic modifications including suppression of *Lgr5* by trimethylation of histone H3K27 and activation of *p21* by DNA demethylation play critical roles during stem cell aging. Organoids derived from aged animals could be a powerful research tool for exploring the molecular mechanisms underlying stem cell aging and for development of anti-aging interventions. Further studies to validate our results using human materials will be essential, and may lead to treatments that can prolong healthy life expectancy.

## Methods

### Establishment of intestinal epithelial organoids

Isolation and dissociation of stem cells from normal intestinal epithelia of wild-type C57BL/6 mice were performed.^3^ Isolated intestinal epithelial stem cells were embedded in Matrigel (growth factor-reduced, phenol red-free; BD Biosciences) and seeded in 48-well plates. The cells were overlaid with 250 μL/well basal culture medium (advanced Dulbecco’s modified Eagle medium/F12 supplemented with penicillin/streptomycin, 10 mmol/L HEPES, Glutamax, 1 × N2, 1 × B27 [all from Thermo Fisher Scientific], and 1 mmol/L *N*-acetylcysteine [Sigma-Aldrich]) containing EGF, noggin, Y-27632, and Rspo1. For this study, we considered mice over 50-weeks-old to be aged mice, and considered establishment of intestinal epithelial organoids to be successful when they could be cultured for over 5 passages. Images were acquired using either a fluorescence microscope equipped with phase-contrast optics (CKX41, Olympus) or the Olympus Fluoview system (FV1000D, Olympus). Before observation, organoids were fixed with 4% paraformaldehyde phosphate buffer solution (Wako) for 30 min and 0.25% Triton X-100 (Sigma) for 15 min at room temperature to increase cellular permeability. DNA was stained with DAPI (Molecular Probes). All experiments and procedures were approved by the Keio University Animal Research Committee, and all methods were carried out in accordance with the approved guidelines.

### Apoptosis assay

To detect apoptotic cell death, the TUNEL assay was performed using an in situ Apoptosis Detection Kit (Takara Bio). TUNEL assay uses terminal deoxynucleotidyl transferase to label 3′-OH ends of DNA fragments that are generated during the process of apoptosis. The cells undergoing apoptosis are specifically labeled with fluorescein-dUTP with high sensitivity, allowing the immediate detection by viewing with a fluorescein microscope.

### Cell proliferation assay

Cell proliferation activity of organoids was evaluated by cell counting using a hemocytometer and WST assay using the Cell Counting Kit-8 (Dojindo) that allows sensitive colorimetric assays for the determination of cell viability. Highly water-soluble tetrazolium salt, WST-8, is reduced by dehydrogenase activities in cells to give a yellow-color formazan dye, which is soluble in the tissue culture media. The amount of the formazan dye generated by the activities of dehydrogenases in cells is directly proportional to the number of living cells.

### Cell cycle assay

Cells were harvested by trypsinization, washed with PBS and fixed in 70% ice-cold ethanol overnight at 4 °C. They were then washed with PBS and treated with RNase A (1 mg/mL) at 37 °C for 60 min and incubated with propidium iodide (50 mg/mL) for 30 min at room temperature. After incubation, flow cytometry analysis was performed using LSRII (BD Biosciences).

### SA-β-gal assay

SA-β-gal is commonly used as a marker of cellular senescence. Evaluation of cellular senescence in organoids was performed using a SPiDER-βGal detection kit (Dojindo) that allows to detect SA-β-gal with high sensitivity. SPiDER-βGal is a reagent to detect SA-β-gal with high cell-permeability and high retentivity inside cells.

### RNA extraction and microarray analysis

Total RNAs from cultured intestinal organoids were extracted using the RNeasy Plus Mini Kit and QIAshredder (QIAGEN). Microarray analysis was conducted by Toray Industries (Tokyo, Japan). In brief, extracted total RNA was checked with a Bioanalyzer (Agilent Technologies) and labeled with Cy5 and Cy3. The labeled RNAs were hybridized onto a Human Oligo chip 25k (Toray Industries). After stringent washing, the fluorescent signals were scanned with a 3D-Gene Scanner (Toray Industries) and analyzed using the 3D-Gene Extraction software (Toray Industries). The raw data for each spot were normalized by subtraction of the mean background signal intensity determined from the signal intensities of all blank spots with 95% confidence intervals. The relative expression level was calculated by comparing the signal intensities of the valid spots throughout the microarray experiments. All data were submitted to the GEO database, under the accession number GSE103634.

### Gene set enrichment analysis (GSEA)

GSEA was performed using the database from version 3.1 of the molecular signature database: the C2 curated gene sets from online pathway databases, PubMed publications and knowledge of domain experts.^30^ The gene sets APC_TARGETS, EMBRYONIC_STEM_CELL_CORE, HALLMARK_MYC_TARGETS and DNA_REPLICATION were used for GSEA.

### Quantitative RT-PCR

Expression levels of *Lgr5* and Wnt signaling genes (*Rnf43, Axin2, c-Myc*), cellular senescence genes (*p21, p16*) and DNA methyltransferase genes (*Dnmt1, Dnmt3a, Dnmt3b*) were analyzed by quantitative RT-PCR using Universal SYBR Select Master Mix (Thermo Fisher Scientific). *Gapdh* was used as an internal control. The primer sequences are summarized in Supplementary Table S1.

### Bisulfite sequencing

Total DNAs from cultured intestinal organoids were extracted using a QIAamp DNA Mini Kit (QIAGEN). Isolated DNA was bisulfite converted and purified using an EpiTect Bisulfite Kit (QIAGEN). Template DNA was amplified using Takara EpiTaq™HS for bisulfite-treated DNA (TaKaRa). The primer sequences are summarized in Supplementary Table S1. DNA libraries were made using a TOPO TA Cloning Kit (Invitrogen) and a QIAprep Spin Miniprep Kit (QIAGEN). We then examined the sequences using an Applied Biosystems genetic analyzer (Thermo Fisher Scientific).

### Chromatin immunoprecipitation (ChIP) assay

Chromatin samples were isolated from organoids using the Rakutippu ChIP Assay Kit (MAB Institute) and fragmented using Bioruptor VCD-250 (Cosmo Bio). Chromatin samples were immunoprecipitated with a mouse monoclonal antibody against trimethylated histone H3 lysine 27 (MAB Institute) at 4 °C overnight. DNA was purified from immunoprecipitated chromatin samples and subjected to real-time PCR. The results of real-time PCR were corrected with input DNA obtained from the same organoids without immunoprecipitation. The primer sequences are summarized in Supplementary Table S1.

### Drug treatment

Intestinal epithelial organoids were treated with 20 μM DZNep (Sigma-Aldrich) and 100 μM NMN (Tokyo Chemical Industry). After drug treatment, gene expression, cell proliferative ability and NAD^+^ level were examined.

### Measurement of NAD^+^ level

NAD^+^ levels in intestinal epithelial organoids were measured using a NAD/NADH assay kit-WST (Dojindo) that allows to determine intracellular amounts of total NAD^+^/NADH and NADH. Intracellular NAD^+^ levels were determined by subtracting NADH levels from total NAD^+^/NADH levels.

### Statistical analysis

Statistical analyses were performed with Fisher’s exact test, *t*-test and two-way ANOVA. Results of 5–8 experiments were plotted as mean ± standard deviation (SD). All comparisons were two-sided, and differences at *p* < 0.05 were considered significant. (*p < 0.05, **p < 0.01, ***p < 0.001).

## Electronic supplementary material

Supplementary Table S1

## Data Availability

Data that support the findings of this study have been deposited in the GEO database with the accession number GSE103634.

## Electronic supplementary material

**Supplementary information** accompanies the paper on the *npj Aging and Mechanisms of Disease* website (10.1038/s41514-018-0031-5).
