# Publisher Correction: Epigenetic silencing of *Lgr5* induces senescence of intestinal epithelial organoids during the process of aging

**DOI:** 10.1038/s41514-019-0035-9

**Published:** 2019-03-07

**Authors:** Ryoei Uchida, Yoshimasa Saito, Kazuki Nogami, Yohei Kajiyama, Yukana Suzuki, Yasuhiro Kawase, Toshiaki Nakaoka, Toshihide Muramatsu, Masaki Kimura, Hidetsugu Saito

**Affiliations:** Division of Pharmacotherapeutics, Keio University Faculty of Pharmacy, 1-5-30 Shiba-kohen, Minato-ku, Tokyo, Japan

**Correction to:** *npj Aging and Mechanisms of Disease* 10.1038/s41514-018-0031-5, Published online 01 December 2018

The original version of this Article had an incorrect Article number of 1, an incorrect Volume of 5 and an incorrect Publication year of 2019. These errors have now been corrected in the PDF and HTML versions of the Article.

