# Supplementary material for: Epigenetic silencing of Lgr5 induces senescence of intestinal epithelial organoids during the process of aging
Source: NPJ Aging Mech Dis. 2018 Dec 1;4:12. doi: 10.1038/s41514-018-0031-5 (PMC6279747; doi:10.1038/s41514-018-0031-5)
Supplement: Supplementary file 1 — Supplementary Table S1 [file 41514_2018_31_MOESM1_ESM.pdf]

Supplementary Table S1 The primer sequences

Gene	Forward	Reverse
Lgr5	5'-GGACCAGATGCGATACCGC-3'	5'-CAGAGGCGATGTAGGAGACTG-3'
Rnf43	5'-CACGAGTTTCATCGAACGTGT-3'	5'-CTGGCGAATGAGGTGGAGT-3'
Myc	5'-ATGCCCCTCAACGTGAACTTC-3'	5'-GTCGCAGATGAAATAGGGCTG-3'
Axin2	5'-ATGAGTAGCGCCGTGTTAGTG-3'	5'-GGGCATAGGTTTGGTGGACT-3'
p16	5'-ATGGAGTCCGCTGCAGACAG-3'	5'-ATCGGGGTACGACCGAAAG-3'
p21	5'-ACTTCCTCTGCCCTGCTGC-3'	5'-GGTCTGCCTCCGTTTTCG-3'
Dnmt1	5'-CCGTGGCTACGAGGAGAAC-3'	5'-TTGGGTTTCCGTTTAGTGGGG-3'
Dnmt3b	5'-CCTGTGGAGTTTCCGGCTAC-3'	5'-GACGCTCTTAGGTGTCACTTC-3'
Sirt1	5'-TGATTGGCACCGATCCTCG-3'	5'-CCACAGCGTCATATCATCCAG-3'
Gapdh	5'-TGTGTCCGTCGTGGATCTGA-3'	5'-CCTGCTTCACCACCTTCTTGA-3'
Lgr5-ChIP	5’-GCGATTTCTTTGAGGCTTTG-3’	5’-ATCCGAAAGATTGGCATCAC-3’
p21-Bisulfite	5’-AAGAGGAGGTTTGTTTAGGTTAGTTAAATT-3'	5’-ACCAATAATTCATCAAAATTACATAACATC-3'
